# Post-traumatic upper cervical subluxation visualized by MRI: a case report

**DOI:** 10.1186/1746-1340-15-20

**Published:** 2007-12-19

**Authors:** James Demetrious

**Affiliations:** 1 Private practice, Wilmington, NC, USA; 2 Post-gradate faculty, New York Chiropractic College, Seneca Falls, NY, USA

## Abstract

**Background:**

This paper describes MRI findings of upper cervical subluxation due to alar ligament disruption following a vehicular collision. Incidental findings included the presence of a myodural bridge and a spinal cord syrinx. Chiropractic management of the patient is discussed.

**Case presentation:**

A 21-year old female presented with complaints of acute, debilitating upper neck pain with unremitting sub-occipital headache and dizziness following a vehicular collision. Initial emergency department and neurologic investigations included x-ray and CT evaluation of the head and neck. Due to persistent pain, the patient sought chiropractic care. MRI of the upper cervical spine revealed previously unrecognized clinical entities.

**Conclusion:**

This case highlights the identification of upper cervical ligamentous injury that produced vertebral subluxation following a traumatic incident. MRI evaluation provided visualization of previously undetected injury. The patient experienced improvement through chiropractic care.

## Background

For many years, chiropractors have utilized x-ray to assess spinal alignment, perform biomechanic assessments and evaluate spinal structures for traumatic and pathologic entities. While x-ray remains invaluable to the practicing chiropractor, new technologies have emerged that provide an improved vantage with regard to the visualization of spinal integrity.

As defined by the Association of Chiropractic Colleges, a subluxation is a complex of functional and/or structural and/or pathological articular changes that compromise neural integrity and may influence organ system function and general health. A subluxation is evaluated, diagnosed, and managed through the use of chiropractic procedures based on the best available rational and empirical evidence [1].

Magnetic resonance imaging provides a nearly unparalleled assessment of the spine. It provides a view of anatomic and physiologic processes while providing a unique opportunity to evaluate patho-physiologic entities. MRI is particularly valuable toward improving the conspicuity and visualization of potential precursors to biomechanic flaws that may adversely affect intervertebral joint mechanics.

Specific to spinal trauma, evidence of injury can be appreciated via MRI. Ligamentous disruption can produce inflammation that can be readily visualized [2,3]. Annular tears and rim lesions described in cadaveric studies by Taylor and Twomey [4] can be visualized as High-Intensity Zones on MRI [5]. Recent studies have evaluated mechanobiologic issues and diffusion patterns that provide marvellous glimpses of the affect of endplate damage and subsequent disc desiccation/degeneration [6].

Images produced by MRI also provide physiologic assessments of adaptability following injury. Intermediate and late stage adverse effects of biomechanic flaws can be readily visualized utilizing MRI. Vertebral body marrow degeneration has been described and classified by Modic [7]. Atrophic changes of paraspinal musculature are readily visualized using MRI [8,9].

In the case presented, a patient involved in a vehicular collision suffered injury to the upper cervical spine that was not detected or appreciated utilizing standard imaging protocols. Additional MRI evaluation of the upper cervical spine provided objective identification of previously undetected ligamentous injury, spinal subluxation, the identification of a myodural bridge and visualization of a spinal cord syrinx. Refined chiropractic care protocols were employed that led to a favourable outcome.

## Case presentation

### Case report

A twenty-one year old female presented with complaints of upper neck and head pain subsequent to a vehicular collision that occurred two days prior. While driving a midsize vehicle, a pickup truck crossed into her lane of traffic. Her vehicle was impacted on the front/left aspect of her car.

The patient reported that she was travelling at a rate of speed of 45 mph. Responding officers estimated that the offending pickup truck was travelling at a speed of 55 mph. Severe vehicular damage occurred mandating a fire department rescue to extricate the patient from the wreckage. The patient was unconscious during the rescue and was transported to a local hospital emergency department. Upon arriving at the hospital, the patient suffered a seizure and subsequently regained consciousness.

The patient was admitted to the hospital and underwent evaluation and imaging of the head and neck. X-rays and CT scan of the head and cervical spine were interpreted as normal. The patient received care for abrasions related to the accident and was released from the hospital. She was advised to follow-up with a medical neurologist. Subsequent neurologic care included a prescription of migraine medication.

Upon presentation to our office, the patient's primary symptoms included sub-occipital neck pain, dizziness and persistent sub-occipital headache. Using pain drawings and visual analogue scales, she indicated that the pain rated 9/10 (0 = No Pain, 10 = The Worst Pain of One's Life). She reported medications prescribed did not provide relief. No other symptoms were reported.

Examination revealed a 6'2", 175 pound athletic caucasian female. She was afebrile, her blood pressure was 122/76 and her pulse was 68 beats per minute. Visual inspection revealed guarded and restricted head and neck motion. Palpation revealed exquisite midline C2 spinous tenderness and decreased compliance of the sub-occipital musculature.

She experienced pain and restricted motion at the cervical-cranial junction on active right cervical rotation (20°), right lateral flexion (5°), flexion (10°) and extension (15°). Careful cervical compression was performed due to increased pain in extension, neutral and flexed postures, causing localized pain to C1/2 with radiation into sub-occipital region of the head. Valsalva manoeuvre produced neck and head pain. Complete neurologic evaluation revealed no apparent abnormalities.

Chiropractic evaluation was performed. Decreased intersegmental motion and fixations were noted affecting CO/1 and C1/2. Thermographic instrumentation revealed asymmetry of heat patterns of the upper cervical spine. Flexion and extension stress x-ray views failed to reveal spinal hypermobility or increase in the Atlanto-Dental Interval that would suggest instability.

**Figure 1:**
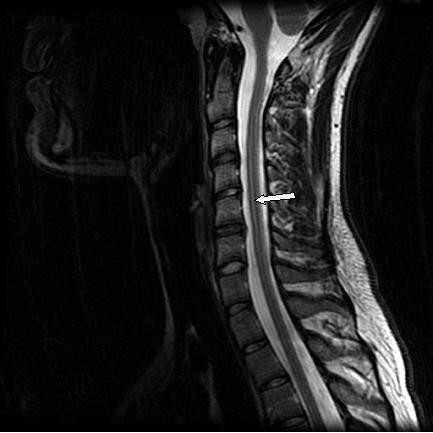
T2 Weighted Image: Syrinx extending from C2-C7 (Large White Arrow).

Due to the mechanism and severity of the patient's collision combined with persistent severe symptoms affecting the upper cervical spine not previously imaged, a high-resolution MRI of Occiput-C7 was ordered. The attending neuroradiologist reported a cervical spinal cord syrinx that extended from C2-C7. (Figure 1). No other abnormalities were noted.

**Figure 2:**
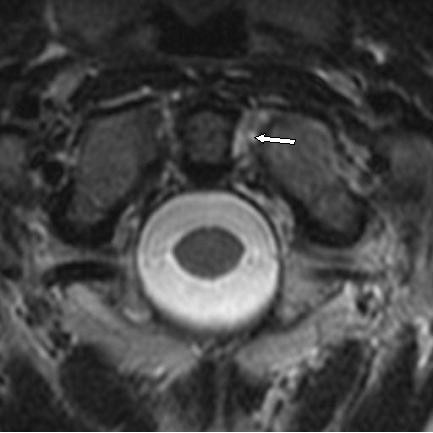
Axial T2 Weighted Image reveals hyperintense signal corresponding to Alar Ligament sprain disruption (White Arrow).

**Figure 3:**
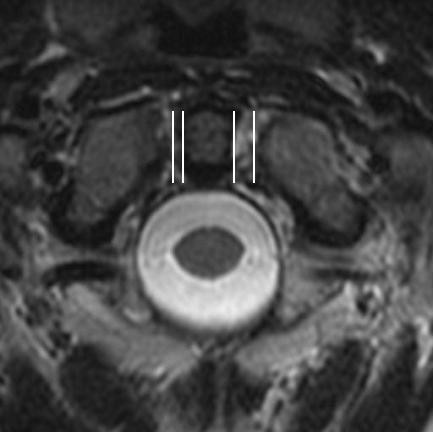
Axial T2 Weighted Image – Translational atlanto-axial subluxation.

Upon over-reading the study in our office, the MRI images revealed left alar ligament disruption as evidenced by increased signal on T2 weighted images (See Figures 2 and 3). Left lateral translational subluxation was visualized. Upon re-evaluation, the neuroradiologist concurred with these opinions, suggested that additional coronal views may provide improved visualization and wrote an addendum to his report.

**Figure 4:**
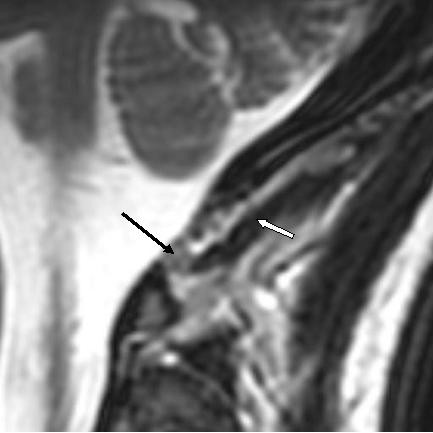
Sagital T2 Weighted Image – Myodural Connection (Black Arrow). Rectus Capitus Posterior Minor (White Arrow).

**Figure 5:**
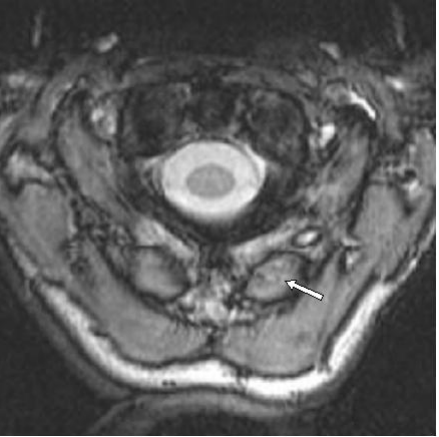
Axial 3D MRI – Rectus Capitus Posterior Minor (Arrow).

An incidental finding included a visualized myodural bridge intervening between the rectus capitis posterior minor (RCPMi) and the spinal cord dura. (Figure 4) A normal appearing RCPMi was visualized on axial views with good margins, composition and cross-sectional area (Figure 5).

The patient's diagnosis included:

• Loss of consciousness, seizure, dizziness and headache.

• Post-traumatic chiropractic spinal subluxations affecting CO/1 and C1/2,

• Alar ligament disruption,

• Strain injuries of the sub-occipital musculature and,

• The presence of a cervical spinal cord syrinx.

Chiropractic care was initiated utilizing specific adjustments targeted to vertebral subluxations of the upper cervical spine in pain-free ranges of motion. Seated Gonstead chiropractic protocols were utilized. Pre-test evaluations were performed to assess reported pain on passive ranges of motion. Due to the presumed injury of the alar ligament, the author selectively avoided pain provocative planes of motion during adjustive procedures. The patient tolerated this well without reported discomfort.

Chiropractic care was rendered at a rate of three visits per week for six weeks. Range of motion and proprioceptive ball exercises of the cervical spine that incorporated vestibulo-occular activities were provided to the patient during the second week of care. Through six weeks of care, the patient reported progressive improvement to 75% symptomatic resolution as evidenced by weekly Pain Drawings and Visual Analogue scale outcome measures. Objective benchmarks including ranges of motion, thermographic readings, postural and palpatory evaluations of muscular compliance improved.

**Figure 6:**
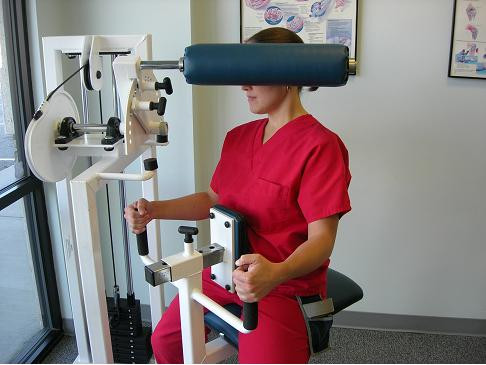
8-Way Neck Isotonic Exercise Rehabilitation.

The patient was progressively tapered from passive care techniques and was provided therapeutic strengthening rehabilitation utilizing 8-Way neck isotonic equipment in pain-free ranges of motion (Figure 6). This equipment provides isotonic strength rehabilitation in cervical flexion, oblique flexion, lateral flexion, oblique extension and extension. Additional home exercises were provided to the patient.

The patient continued active care for a period of four weeks at three visits per week while reducing passive care modalities. The patient reported 100% symptomatic improvement at the conclusion of care. At six months follow-up, the patient remained asymptomatic.

## Discussion

### Literature review

The patient in this case suffered cervical acceleration/deceleration (CAD) Grade III injury. As described by Croft, a CAD Grade III injury represents a moderate severity injury with associated limitation of motion, ligamentous instability and neurologic findings [10]. The utilization of MRI of the upper cervical spine helped to objectively define the presence of ligamentous involvement.

Undiagnosed spinal trauma can significantly impair biomechanic function. Core ligamentous, disk, endplate, zygapophyseal, muscular and neural tissue injuries produce significant prognostic complications as evidenced by the following studies:

Uhrenholt et al. reported subtle lesions found exclusively in MVA victims included annular fibrosis tears, disc disruption with herniation, avulsions/separations between the endplate and vertebra, articular cartilage microfractures, hemarthrosis, capsular swelling or bruising, new vertebral fractures, bruising of synovial folds. They concluded that negative clinical and radiologic exam do not prove the absence of patho-anatomical lesions [11].

Panjabi reported soft tissue injuries associated with whiplash often may not be visualized on routine radiographs or CT scans. Soft tissues involved in low velocity whiplash seldom tear completely and are often stretched beyond the elastic limits, resulting in incomplete injuries [12].

In cadaveric studies, Taylor and Twomey demonstrated undiagnosed disc rim lesions, facet capsular tears and zygapophyseal articular fractures not appreciated through x-ray evaluation [4]. Kaplan et al. report that visualized annular tears termed, "High Intensity Zones," represent linear fissures through all or part of the disc annulus. They report that nerve ingrowth from the surface of the disc may lead to pain [13].

Ito reported chronic pain resulting from low-speed collisions may be explained by partial tears of soft tissues including annular fibers, ligaments and avascular cartilage. Because of poor blood supply, these tissues may not completely heal following injury. Resulting injuries produce altered cervical spine kinematics that can lead to accelerated degenerative changes and clinical instability [14].

Spinal ligaments are readily visualized utilizing MRI. High resolution T2 weighted images have been shown to reliably provide evidence of spinal ligament, capsular and muscular trauma as evidenced by increased signal intensity that corresponds to acute inflammation. Benedetti and Krakenes provide MRI evidence of alar ligament disruption as evidenced by signal hyperintensity and subluxation [2,3].

Conflicting studies exist that questions the reliability of increased T2 signal in the region of the alar ligament visualized on MRI. Roy et al. reported increased signal in the region of the alar ligament in one third of the ligaments evaluated in fifteen asymptomatic subjects [15]. Pfirrmann reported asymmetric high signal intensity of the alar ligament in the majority of non-injured cases [16]. However, Krakene points out that Roy and Pfirrmann's findings may not be accurate due to inadequate imaging protocols, the use of a small magnet (0.5 Tesla) and poor image quality.

Regarding care related to whiplash associated disorders, Rosenfeld reported that active intervention was more beneficial than rest protocols [17]. Sowa et al. reported promising clinical evidence continues to accumulate for the effectiveness of motion-based therapies in the treatment of low back pain. Their results demonstrate the anti-inflammatory and protective effect of tensile force on the annulus of the intervertebral disc, suggesting that motion can be beneficial to inflamed cells [18].

The existence of the cervical myodural bridge was originally established by Hack et al. The relationship of this anatomic entity and its relationship to cervicogenic headache have been documented [19]. Hack has hypothesized that exertion through the myodural bridge may exert tension through the pain sensitive dura. Furthermore, he indicates that chiropractic adjustive procedures likely prove beneficial through this anatomic relationship.

Hallgren has demonstrated the effect of injury and denervation in the genesis of atrophic and fatty infiltrated changes of the Rectus Capitus Posterior Minor on MRI [20]. Elliott et al. reviewed the relationship of paraspinal core muscle atrophic changes following spinal dysfunction [21].

The development, timing and etiology of post-traumatic syrinx development are often unknown. Trauma has been implicated. The onset of new symptoms in a patient who has already sustained significant cord injury can be catastrophic and devastating [22].

### Diagnostic considerations

In assessing post-traumatic spinal conditions, it is requisite to image the site of pain. The literature reflects the improving resolution of MRI and its ability to image and classify ligamentous injuries. If patients present with upper cervical complaints, it is essential, based upon the patient's clinical presentation, to image acute or previously undetected injuries that may play a role in pain genesis, degenerative changes and potential instability.

The American College of Radiology (ACR) reports that imaging of patients with suspected cervical spine trauma is one of the most controversial topics in medicine today. The problem is not merely one of radiology, but touches all spinal sub-specialties. The ACR considered several questions: 1) which patients need imaging, 2) how much imaging is necessary, and 3) exactly what sort of imaging is to be performed, and developed the ACR Appropriateness Criteria™ Guidelines [23].

The ACR Appropriateness Criteria™ Guidelines included the initial investigations of 5,719 patients with cervical trauma. The literature review for this revision included data on 13,534 patients. In addition, they utilized data drawn from the National Emergency X-Radiography Utilization Study (NEXUS) of 34,069 patients and from the Canadian Rule group of 8,924 patients.

Although the literature still recommends flexion/extension radiographs, it was the opinion and experience of the ACR panel that they are not very helpful, particularly in the acute trauma setting. They suggested that flexion/extension radiography is best reserved for follow-up of symptomatic patients, usually in 7 to 10 days after muscle spasm has subsided. As such, MRI plays an important role in the determination and assessment of ligamentous integrity. The ACR reports that there is agreement in the literature that MRI is most appropriate for adult patients with suspected spinal trauma:

• Who are alert with cervical tenderness, with paresthesias in hands or feet;

• Who are unconscious;

• Who exhibit impaired sensorium >48 hours (alcohol and/or drugs) with or without neurologic findings;

• Who have neck pain, clinical findings suggestive of ligamentous injury, radiographs and/or CT "normal.";

• Who have clinical evidence of spinal cord injury.

When ordering MRI studies of the cervical spine, it is important to recognize the typical protocols utilized by imaging centers often include axial view images of C2-C7. Kaplan et al. describe a cervical spine protocol for image acquisition that includes obtaining images from C2-3 through C7-T1. They note that variations of spine protocols may work easily as well [13]. This beckons the question, if the upper cervical spine is not being evaluated on a regular basis, what injuries have been left undetected?

### Therapeutic considerations

In this case, chiropractic adjustments were provided to an upper cervical spine that showed evidence of alar ligament disruption. Was this prudent and did the imaging findings provide sufficient evidence to constitute an absolute contraindication to forces generated during chiropractic adjustment?

Flexion/extension x-ray views of the cervical spine did not reveal segmental instability. Typical MRI protocols failed to adequately image the upper cervical spine [13]. As such, it is possible that practitioners are providing spinal care to undetected injured alar ligaments unbeknownst to them. After correlating the results of a high-resolution MRI study of the upper cervical spine with clinical findings, treatment was provided in this case with a refined understanding of the clinical picture.

The decision making process to provide chiropractic adjustment to a presumed alar ligament injury was made based upon the overwhelming evidence that supports the therapeutic benefit of motion based therapies. Spinal articular structures are dependent upon movement during healing to re-establish and promote segmental motion, structural integrity, alignment of scar tissue along stress planes, improve proprioception, synovial and lymphatic fluid drainage, disc and cartilage health [24,25].

Impaired motion clearly has a detrimental effect that leads to degenerative sequelae. The literature clearly defines the effect of immobilization of joints and its adverse effect through atrophy of regional musculature. In addition, the restorative effect of renewed mobility and exercise on previously immobilized and atrophic muscle tissue is well-documented [26]. Paraspinal muscles exert control through segmental and global effect [27]. In an article by Mayer et al. MRI of patients following lumbar extension exercises produced hyperintense signals that corresponded to post-exercise perfusion and blood pooling within the paraspinal musculature [28].

Empirically, chiropractors restore segmental motion to spinal structures. In this case, the author identified potential ranges of motion that may have been suspect for weakness and instability. As a prudent clinician would not apply a painful valgus force to an acutely injured medial collateral ligament, the author selected ranges of motion that did not exacerbate the upper cervical spine and unduly stress injured planes of motion. In doing so, chiropractic care was safely provided to the patient and a positive outcome was achieved.

### Clinical relevance

With regard to MRI, issues of clinical relevance are important considerations. Does visualized hyperintensity on T2 weighted images correlate to the patient's symptoms? If inflammation is present affecting the alar ligament, intervertebral disk or capsular ligaments, how does this additional hydrostatic fluid burden affect tissue health? Clearly, MRI must be correlated to other aspects of patients' clinical presentations.

## Conclusion

MRI provided objective evidence of upper cervical ligamentous injury and components of chiropractic subluxation were demonstrated that led to a refined approach and a favourable outcome. In this case, chiropractic care was carefully applied and led to the resolution of acute neck pain, associated headache and dizziness secondary to post-traumatic subluxation.

Clinicians must realize that typical cervical spine MRI protocols may not include adequate visualization of CO/C1/C2. Ligamentous injuries may be missed if imaging is not requested of the upper cervical spine. Clinicians should consider requesting additional high-resolution MRI protocols that include occiput-C3. Additional coronal views may provide improved visualization of these structures.

More study to evaluate issues related to the stability/instability of C0/C1/C2 could be undertaken utilizing an upright MRI scanner during cervical flexion, extension, rotation and lateral flexion. Through careful correlation of MRI findings to clinical manifestations, perhaps improved clinical relevance of presumed ligamentous injury can be achieved.

MRI is an important imaging modality that provides objective evidence of spinal ligamentous injury. It provides visualization of potential precursors to biomechanic flaws that may adversely affect intervertebral joint mechanics. Further scientific investigation is needed to evaluate the role of MRI in chiropractic practice. This unique technology may have the capability of visualizing diagnostic considerations and restorative processes of healing inherent to chiropractic intervention.

## Competing interests

The author(s) declare that they have no competing interests.
